# Endoscopic Management of Perforation of Right Hepatic Duct Following Non-Surgical Abdominal Trauma

**DOI:** 10.1155/1997/93048

**Published:** 1997

**Authors:** B. C. Sharma, A. Maini, V. A. Saraswat

**Affiliations:** 1 Departments of Gastroenterology Sanjay Gandhi Postgraduate Institute of Medical Sciences Lucknow India; 2 Departments of Nuclear Medicine Sanjay Gandhi Postgraduate Institute of Medical Sciences Lucknow India

## Abstract

Isolated bile duct injuries after blunt abdominal trauma are rare. Surgery is the usual mode of treatment. We report a patient with a right hepatic duct injury following blunt abdominal trauma who was managed successfully by endoscopic papillotomy.


*HPB Surgery*, 1997, Vol. 10, pp. 245–247
Reprints available directly from the publisher
Photocopying permitted by license only

© 1997 OPA (Overseas Publishers Association)
Amsterdam B.V. Published in The Netherlands
by Harwood Academic Publishers
Printed in India

### *Case Report*

### Endoscopic Management of Perforation of Right Hepatic Duct Following Non-Surgical Abdominal Trauma

B. C. SHARMA[a], A. MAINI[b] and V. A. SARASWAT[a]

*Departments of Gastroenterology [a] and Nuclear Medicine [b], Sanjay Gandhi Postgraduate Institute of Medical Sciences, Lucknow (India)*

*(Received 17 January 1996)*

**Isolated bile duct injuries after blunt abdominal trauma are rare. Surgery is the usual mode of treatment. We report a patient with a right hepatic duct injury following blunt abdominal trauma who was managed successfully by endoscopic papillotomy.**

*Keywords:* Bile duct, Biliary tract, Bile duct injury, Biliary fistula, Bile leak, Sphincterotomy

### INTRODUCTION

Biliary tract injuries occur in 1–5% of cases following abdominal trauma [1–4]. Over 80% of such injuries are caused by penetrating trauma [2, 4–6]. Blunt abdominal trauma accounts for a minority of cases. About 80–85% of such injuries are sustained by the gallbladder whereas injury to the bile duct occurs in 15–20% of cases [5, 6]. Such cases commonly have other associated injuries and invariably require surgical management. We report a case of isolated right hepatic duct injury following blunt abdominal trauma, which was managed endoscopically.

### CASE REPORT

A 35 year old man presented following a fall from a height with his abdomen striking the ground. He remained well until three days late when he noted gradual distension of his abdomen. He had low grade fever but no jaundice. Physical examination revealed a febrile (38°C), ill looking man without jaundice. The abdomen was distended and there was mild diffuse tenderness. There was no organomegaly and bowel sounds were normal.

Laboratory investigations revealed hemoglobin 10.6 g/dl, total leucocyte count $11.5 \times 10^9$/l, total bilirubin 34 μmol/l, alkaline phosphatase 200 IU/l, AST 56 IU/l and ALT 36 IU/l. Chest radiograph was normal. Abdominal ultrasound revealed free fluid in the abdomen with some loculated collections. Peritoneal fluid was bile stained with a bilirubin level of 1530 μmol/l. Hepatobiliary scintigraphy confirmed a leak from the right hepatic duct (Fig. 1). An ERCP revealed the common bile duct and common hepatic ducts to

Correspondence to: Dr. V.A. Saraswat, Department of Gastroenterology, Sanjay Gandhi Post Graduate Institute of Medical Sciences, Raebareli road, Lucknow 226 014 (India).

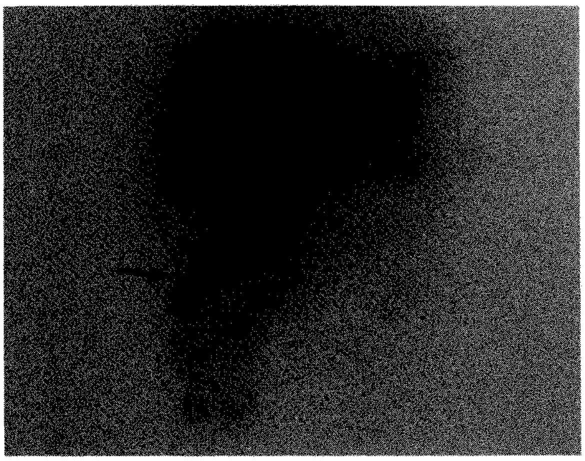

FIGURE 1 99 m-Tc mebrofenin hepatobiliary scan showing extravasation of radionuclide activity (arrow).

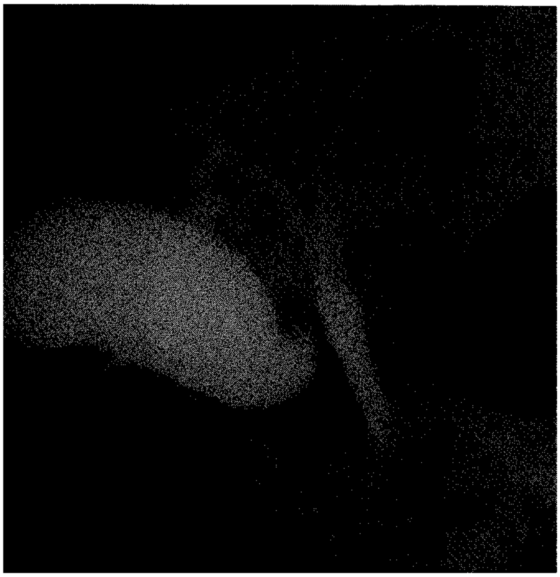

FIGURE 2 ERCP picture showing leakage of contrast from right hepatic duct (arrow).

be normal. There was a leak of contrast from the right hepatic duct (Fig. 2). Endoscopic papillotomy was performed. The abdominal distension gradually subsided and repeat hepatobiliary scintigraphy performed seven days later confirmed closure of the biliary leak . The patient remains asymptomatic after more than one year.

### DISCUSSION

Our patient presented with bile ascites due to right hepatic duct injury following blunt abdominal trauma which could be managed successfully with endoscopic papillotomy.

Biliary tract injuries following abdominal trauma may be diagnosed incidentally at laparotomy. These injuries have significant morbidity from fistula and stricture formation and are associated with a mortality of 10% from other organ injuries [4,5]. Biliary injuries following blunt abdominal trauma are uncommon. The gallbladder and extrahepatic bile duct are protected from blunt abdominal trauma by the rib cage, liver and surrounding viscera. Usually, forceful direct trauma or rapid shearing forces are required to damage the gallbladder and biliary tract [4]. The mechanisms leading to bile duct injuries are not clear. Multiple factors, including shearing force as the liver moves in a cephalic direction, relative rigidity of the bile ducts, rapid emptying of the gallbladder and direct force applied to the duct are thought to be responsible [4].

The right hepatic duct is a relatively unusual site for perforation following blunt abdominal trauma. Bile duct injuries secondary to abdominal trauma commonly occur at the sites where the common bile duct enters the pancreas and where biliary confluence exits from the liver. These sites are points of maximum fixation and are more prone to injury [1–5,7,8].

Our patient presented 72 hours after trauma. Such delayed presentation is known and occurs in 53% of cases [9]. Early presentation (shock and acute abdomen) and late presentation (obstructive jaundice) acccount for the remainder of cases [4]. Delay ranging from a few hours to 60 days, has been reported between clinical presentation and surgical intervention for isolated bile duct injury [7]. Delayed presentations includes symptoms like jaundice, abdominal distention, abdominal pain and fever, due to extravasation of bile in the peritoneal cavity, which were all seen in our patient.

Patients with an acute presentation usually require emergency laparotomy for associated vascular and other visceral injuries. Patients with delayed presentation require intravenous fluids, antibiotics and analgesics and later surgery. The choice of operation depends on the type of biliary injury. For hepatic duct injuries different surgical techniques employed are enteric anastomosis, primary repair, ligation or hepatic resection [4, 10]. Hepaticodochojejunostomy is the preferred surgical technique. Nowadays, endoscopic management has an established role in the treatment of post operative bile duct injuries and spontaneous perforation of the bile duct [11–15]. In partial bile duct injuries following cholecystectomy, endoscopic techniques such as endoscopic papillotomy, biliary stenting and endoscopic nasobiliary drainage have been used successfully [11]. However to the best of our knowledge there are no reports on endoscopic management of biliary tract injury following abdominal trauma.

In the present case, endoscopic papillotomy proved to be a safe and effective treatment. Like post-operative biliary injuries, partial bile duct injuries following blunt abdominal trauma can also be managed endoscopically. However, this modality of treatment is suitable for patients with delayed or late presentation and when there is no evident injury to vascular structures and other viscera.

### *References*

[1] Soderstrom, C.A., Maekawa, K., Du Priest, R.W. Jr. and Cowley, R.A. (1981). Gallbladder injuries resulting from blunt abdominal trauma: an experience and review. *Ann Surg.*, **193**, 60–6.

[2] Posner, M.C. and Moore, E.E. (1985). Extrahepatic biliary tract injury: operative management plan. *J Trauma*, **25**, 833–7.

[3] Mc Nabney, W.K., Rudek, R. and Pemberton, L.B. (1990). The significance of gallbladder trauma. *J Emerg Med.*, **8**, 277–80.

[4] Parks, R.W. and Diamond, T. (1995). Non-surgical trauma to the extrahepatic biliary tract. *Br J Surg.*, **82**, 1303–10.

[5] Kitahama, A., Elliott, L.F., Overby, J.L. and Webb, W.R. (1982). The extrahepatic biliary tract injury: perspective in diagnosis and treatment. *Ann Surg.*, **196**, 536–40.

[6] Bade, P.G., Thompson, S.R., Hirshberg, A. and Robbs, J.V. (1989). Surgical options in traumatic injury to the extrahepatic biliary tract. *Br J Surg.*, **76**, 256–8.

[7] Bourque, M.D., Spigland, N., Bensoussan, A.L., Garel, L. and Blanchard, H. (1989). Isolated complete transection of the common bile duct due to blunt trauma in a child, and review of the literature. *J Pediatr Surg.*, **24**, 1068–70.

[8] Dawson, D.L., Johansen, K.H. and Jurkovich, G.J. (1991). Injuries to the portal triad. *Am J Surg.*, **161**, 545–51.

[9] Michelassi, F. and Ranson, J.H.C. (1985). Bile duct disruption by blunt trauma. *J Trauma*, **25**, 454–7.

[10] Wang, C.H., Lin, R.C., Mo, L.R., Yau, M.P. and Tsai, C.C. (1995). Spontaneous perforation of the left hepatic duct—A case report. *Hepatogastroenterol*, **12**, 77–9.

[11] Saraswat, V.A., Choudhuri, G. and Sharma, B.C., *et al.* (1996). Endoscopic management of post-operative bile leak. *J Gastroenterol and Hepatol*, **11**, 148–151.

[12] Foutch, G.P., Harlan, J.R. and Hoefer, M. (1993). Endoscopic therapy for patients with a postoperative biliary leak. *Gastrointest Endosc.*, **39**, 416–21.

[13] Roblin, D. and Karanjia, N.D. (1994). Endoscopic management of spontaneous perforation of an intrahepatic bile duct. *Endoscopy*, **26**, 751.

[14] Davids, P.H.P., Rauws, E.A.J., Tytgat, G.NJ. and Huibregtse K. (1992). Postoperative bile leakage: Endoscopic management. Gut., **33**, 1118–22.

[15] Binmoeller, K.F., Katon, R.M. and Shneidman, R. (1991). Endoscopic management of postoperative biliary leaks: Review of 77 cases and report of two cases with biloma formation. *Am J Gastroenterol*, **86**, 227–31.

